# Hypertensive disorders of pregnancy (HDP) mortality in the United States: a CDC WONDER population analysis, 1999–2023

**DOI:** 10.3389/frph.2026.1816821

**Published:** 2026-05-20

**Authors:** Macy Anderson, Meghan Fries, Kennedy Haase, Olivia Anne Foley, Abubakar Tauseef

**Affiliations:** 1 Creighton University School of Medicine, Omaha, NE, United States; 2 Hospital Medicine Service Line, CHI Health, Omaha, NE, United States

**Keywords:** age-adjusted mortality rate, CDC WONDER, health disparities, hypertensive disorders of pregnancy, maternal mortality

## Abstract

**Introduction:**

Hypertensive disorders of pregnancy (HDP) are a leading cause of maternal morbidity and mortality in the United States (US), yet national trends across demographic groups are not well characterized.

**Methods:**

This population-based retrospective study analyzed HDP-related maternal mortality from 1999 to 2023 using the Centers for Disease Control and Prevention, Wide-Ranging Online Data for Epidemiologic Research (CDC WONDER). Age-adjusted mortality rates (AAMRs) were calculated using the 2000 US standard population, and trends were evaluated using Joinpoint regression to calculate Annual Percent Change (APC) and Average Annual Percent Change (AAPC) for different demographic groups.

**Results:**

From 1999 to 2023, 1,851 HDP-related deaths occurred. While the AAMR remained stable at 0.10 deaths per 100,000 people, Joinpoint analysis identified a significant upward trend (AAPC 0.89*). AAMR for Non-Hispanic (NH) Black females was 4–5 times higher than the rate for NH White females and increased throughout the study period. Meanwhile, AAMR for NH White women showed no statistically significant overall trend. Age-stratified analysis demonstrated declining mortality among women aged 25–34 (AAPC −1.24*) and a non-significant upward trend in mortality among women aged 35–44 (AAPC 0.24), with rates between the two groups converging from approximately 2018 onwards. Geographic analysis indicated the highest HDP-related mortality burden in the South census region and no significant changes in urban populations.

**Discussion:**

These findings emphasize persistent disparities and the need for equitable access to obstetric and cardiovascular care, as well as improved peri-and postpartum monitoring to reduce preventable maternal deaths. However, these findings should be interpreted in the context of limitations inherent to the use of a single database (CDC WONDER), which may be affected by incomplete reporting in death certificate data.

## 1 Introduction

Hypertensive disorders of pregnancy (HDP) affect between 5%–10% of pregnant women and are the leading cause of pregnancy-related death in the United States (US) (1, 2). HDPs are characterized by elevated blood pressure during pregnancy, either because of the pregnancy or due to pre-existing chronic hypertension. HDPs resulting from pregnancy include gestational hypertension, pre-eclampsia, eclampsia, and pre-existing hypertension complicating pregnancy. These conditions can lead to adverse effects for both the mother and fetus, including required labor induction, placental abruption, stroke, and death in the case of the mother. They can also result in premature birth and low birth weight in the case of the fetus (1). The risk of these complications is reduced by early diagnosis and treatment before, during, and after pregnancy. Importantly, following pregnancy HDP are associated with an increased risk of developing chronic hypertension and future cardiovascular disease, highlighting their role as an early indicator of long-term maternal cardiovascular health (3). The prevalence of HDP has increased in the US from 10.8% to 13.0% between 2017 and 2019, with a 25% increase over the past two decades (1, 2). HDPs disproportionately affect females of color, predominantly Non-Hispanic (NH) Black and American Indian/Alaska Native women, who are more likely to develop mild to severe pre-eclampsia or enter pregnancy with chronic hypertension. Additionally, these females experience the highest ratio of deaths per birth at 41 and 30 pregnancy-related deaths per 100,000 births, respectively, compared to NH White females with 13 deaths for every 100,000 births (4).

Despite improvements in prenatal care, blood pressure monitoring, and antihypertensive therapy, rates of severe complications and death due to HDP continue to rise (5, 6). Existing literature has largely focused on hospital-based outcomes during delivery, highlighting the increasing burden of HDP in the US. However, these studies primarily capture inpatient data and therefore do not reflect population-level mortality trends or deaths occurring outside the hospital setting (7, 8). Because the risks of HDP persist beyond hospital discharge and into the postpartum period, hospital datasets may underestimate these deaths (2, 9). Thus, comprehensive population-based studies are needed to capture deaths throughout the postpartum period and to better characterize the disparities in HDP maternal deaths across race, age, and geography at a national level. Population-based mortality datasets, such as the Centers for Disease Control and Prevention Wide-ranging Online Data for Epidemiologic Research (CDC WONDER) database, capture deaths recorded on death certificates. This data may include deaths occurring during the postpartum period when HDP is listed as an underlying or contributing cause.

To address these gaps, this study analyzes temporal patterns in maternal mortality associated with HDP stratified by race, age, and geographic region in the US from 1999 to 2023. Insight into these trends is essential for informing targeted interventions and equitable health policies to reduce preventable maternal deaths nationwide.

## 2 Methods

The CDC WONDER database was used to identify maternal deaths from HDP occurring within the US (9). Specifically, the “Multiple Cause of Death” option within CDC WONDER was used, allowing identification of HDP listed as either an underlying or contributing cause of death. The Multiple Cause-of-Death Public Use Record and the CDC WONDER database were analyzed to determine HDP as an underlying or contributing cause of death on nationwide death certificate records. This database was previously used to study patterns of pregnancy-related death in the US, where HDP represented one of several cause-specific groups evaluated in the analysis (9). HDP-related mortality was identified using the International Classification of Diseases, 10th Revision, Clinical Modification codes O10-O16 in women aged 25–44, as this age range corresponds to when pregnancy most likely occurs, and aligns with the average US maternal age of 29.6 years (10). Deaths were identified based on ICD-10 codes for HDP listed as an underlying or contributing cause of death, regardless of timing relative to delivery. The study was exempt from institutional review board approval, as the CDC WONDER database contains anonymized, publicly available data.

Data regarding HDP-related deaths and population sizes was extracted from the database from 1999 to 2023. Data on demographic and regional groups was collected, including race/ethnicity, age, urban-rural classification, and region. Racial/ethnic groups were defined as NH White, NH Black or African American, NH American Indian or Alaska Native, NH Asian or Pacific Islander, and Hispanic or Latino people as identified on death certificates. However, only NH White and NH Black or African Americans were included in the analysis, as mortality counts for the other racial/ethnic groups were frequently suppressed or classified as unreliable in CDC WONDER, preventing stable annual rate estimates (9). Age groups were defined as 25 to 34 and 35 to 44 years of age to focus on women of reproductive age, defined as those aged 15–44 (11). Within this broader reproductive age range, women aged 25–44 account for most births in the US, making these age groups particularly relevant for analysis of pregnancy-related outcomes (12, 13). For urban-rural classifications, the National Center for Health Statistics Urban-Rural Classification Scheme was used to divide the population into urban (including metropolitan areas with population >50,000) and rural (non-metropolitan areas with population <50,000). Trends could not be determined for urban populated zones after 2020 due to changes in geographic coding standards, and for rural populated zones from 1999 to 2023 due to unreliable data within the study parameters (9). Regions were classified into Northeast, Midwest, South, and West according to the Census Bureau definitions (14). Due to data suppression, annual mortality from 1999 to 2023 could not be reliably evaluated for the Northeast, Midwest, and West census regions (9). To address data suppression in subgroup analyses, mortality data for groups with high yearly suppression were extracted using aggregated multi-year intervals (1999–2020 and 2021–2023) rather than single-year data.

HDP-related crude and age-adjusted mortality rates were calculated. Age-adjusted mortality rate (AAMR), defined as the number of deaths per 100,000 people, was standardized using the 2,000 United States standard population (15, 16). Population-based rates were used rather than rates per 100,000 live births for two reasons. First, CDC WONDER calculates age-adjusted mortality rates using U.S. Census Bureau July 1 resident population estimates as the denominator for all non-infant age groups; live births are only available as a denominator when querying infant mortality (i.e., deaths under 1 year of age) and cannot be selected for adult age groups. Second, ICD-10 codes O10–O16 capture HDP-related deaths occurring across the antepartum period, delivery, and puerperium, including deaths in pregnancies not resulting in a live birth. A live births denominator would therefore exclude a portion of the at-risk population and introduce systematic underestimation of mortality burden. Use of total female population as the denominator is consistent with population-level mortality surveillance studies using CDC WONDER for obstetric conditions. Numerator (death counts) and denominator (female population estimates) by year are reported. The Joinpoint Regression Program (Joinpoint version 5.4.0 available from the National Cancer Institute, Bethesda, Maryland) was used to determine trends in mortality within the study period (17). Annual percentage change (APC) and average annual percent change (AAPC) with 95% confidence intervals (CIs) for the AAMRs were calculated for the line segments linking a Joinpoint using the Monte Carlo permutation test (18–20). APCs and AAPCs were considered increasing or decreasing if the slope describing the change in mortality over the time interval was significantly different from zero using a two-tailed *t*-test. Statistical significance was set at *p* ≤ 0.05. Asterisks, “*”, were used to denote significance.

## 3 Results

**Figure 1:**
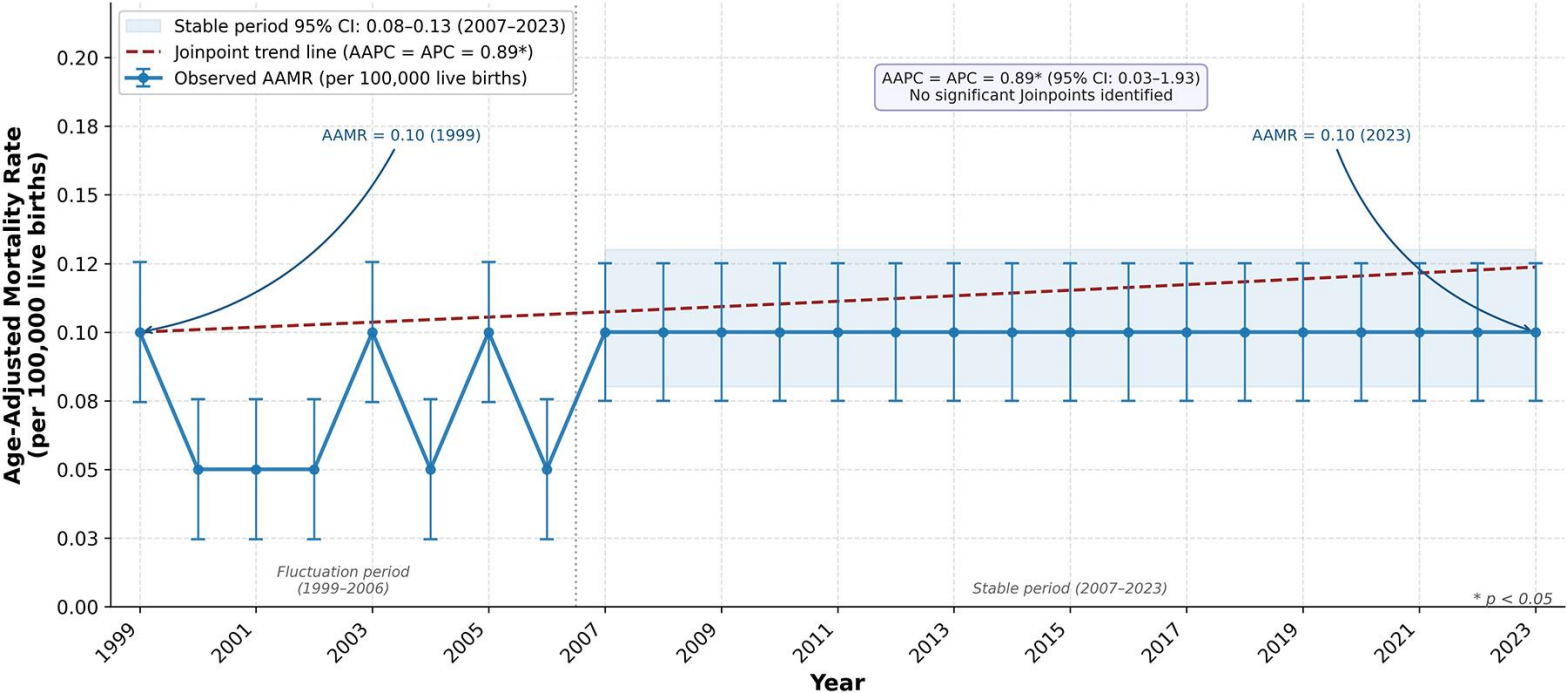
National Age-adjusted mortality rate from hypertensive disorders of pregnancy, United States, 1999–2023. Rates are presented per 100,000 population and age-adjusted to the 2,000 US standard population. Error bars represent 95% confidence intervals. The dashed line represents the Joinpoint trend (AAPC = 0.89*, 95% CI 0.03–1.93). * *p* < 0.05. Values are rounded to two decimal places as returned by CDC WONDER. AAMR, age-adjusted mortality rate; AAPC, average annual percent change; APC, annual percent change; CI, confidence interval.

From 1999 to 2023, there were 1,851 deaths related to HDP in the US. While death rates fluctuated between 0.05 and 0.10 from 1999 to 2006, they stabilized at approximately 0.10* (95% CI 0.08 to 0.13) from 2007 through the remainder of the study period, with minor year-to-year variation not apparent due to rounding to two decimal places, as reported by the CDC WONDER database (Figure 1, Supplementary Table S1). Although absolute values appear numerically small and relatively stable, this reflects the rarity of HDP-related mortality at the population level. Overall, both the AAPC and APC were 0.89* (95% CI 0.03 to 1.93). The Joinpoint analysis did not identify any statistically significant Joinpoints between 1999 and 2023, indicating a single, continuous temporal trend. Consequently, the APC and AAPC are the same, reflecting a statistically significant increase over the study period overall. The steady upward trend suggests a persistent rise in HDP-related mortality over time without evidence of meaningful inflection points (Figure 1).

### 3.1 Race stratified results

**Figure 2:**
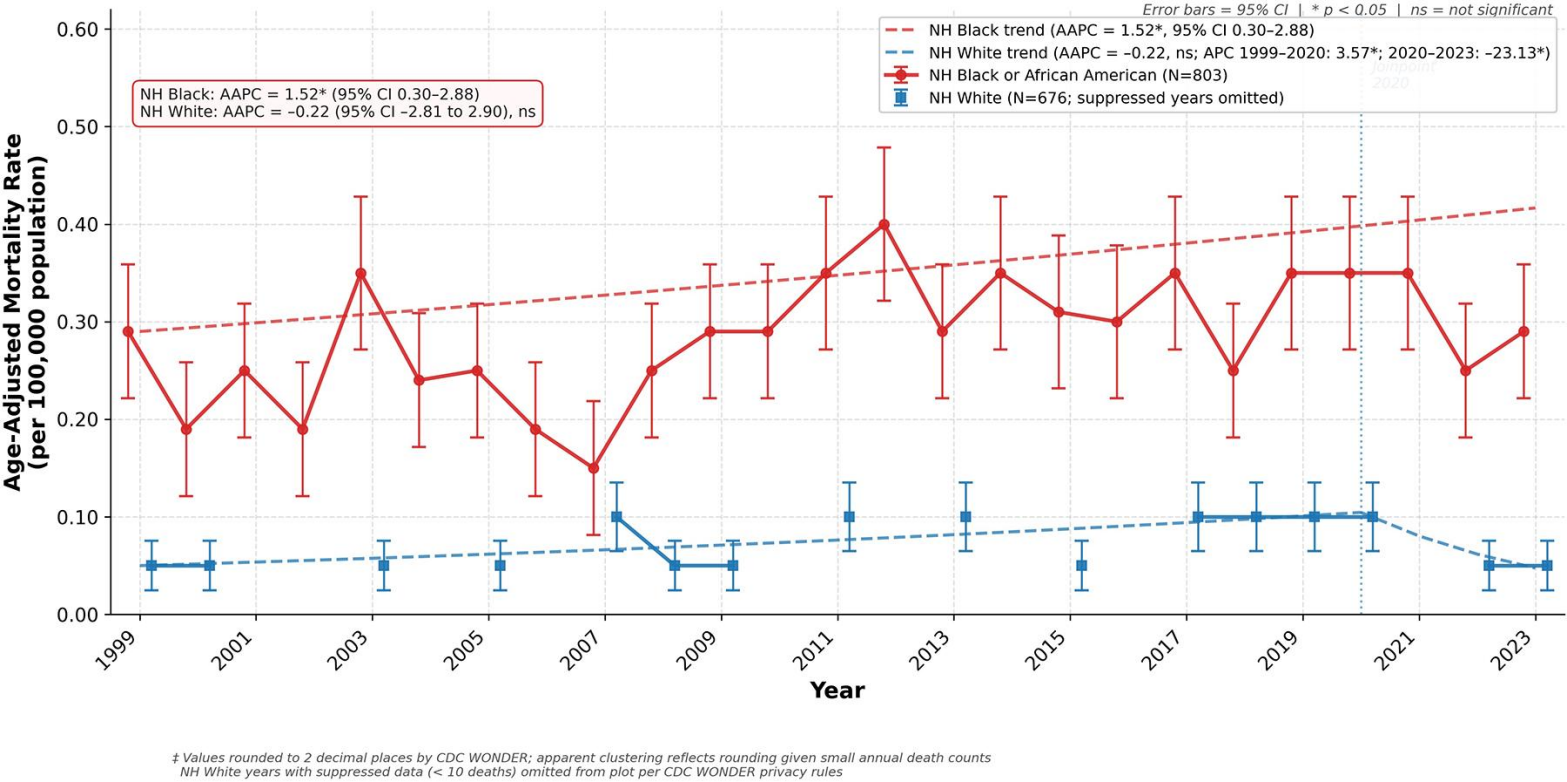
Age-Adjusted mortality rate from hypertensive disorders of pregnancy by race, United States, 1999–2023. Rates are presented per 100,000 population and age-adjusted to the 2,000 US standard population. Error bars represent 95% confidence intervals. The dashed lines represent Joinpoint trends: NH Black or African American (AAPC = 1.52*, 95% CI 0.30–2.88); NH White (AAPC = −0.22, 95% CI −2.81 to 2.90, ns; APC 1999–2020: 3.57*, APC 2020–2023: −23.13*). Years with suppressed data (<10 deaths) are omitted per CDC WONDER privacy rules. * *p* < 0.05. ns = not significant. Values are rounded to two decimal places as returned by CDC WONDER. AAMR, age-adjusted mortality rate; AAPC, average annual percent change; APC, annual percent change; CI, confidence interval.

There were 803 HDP-related deaths among the NH Black or African American population and 676 deaths among the NH White population throughout the study period. The NH Black or African American population had the highest AAMR, ranging from 0.15* (95% CI 0.09 to 0.22) in 2007 to 0.29* (95% CI 0.19 to 0.37) in 2023, with an APC and AAPC of 1.52* (95% CI 0.30 to 2.88). The NH White population had an overall lower and more consistent AAMR throughout the study period, of 0.05* (95% CI 0.03 to 0.07) (Figure 2). Due to relatively low event counts, year-to-year estimates for this group were subject to greater variability, and multiple years were suppressed per CDC WONDER privacy rules, which may limit the stability and interpretability of temporal trend analyses. Rates initially rose between 1999 and 2020 (APC 3.57*, 95% CI 1.46 to 10.09), followed by a sharp decline from 2020 to 2023 (APC −23.13*, 95% CI −38.36 to −0.56). Across the entire study period, the overall trend for NH White individuals was not statistically significant (AAPC −0.22, 95% CI, −2.81 to 2.90) (Supplementary Figure S2). Years with unreliable data were removed from the analysis (Supplementary Table S2).

Although yearly trends could not be determined for the NH American Indian or Alaska Native, NH Asian or Pacific Islander, and Hispanic or Latino populations due to unreliable data, we were able to estimate mortality burden using aggregated multi-year data extracted directly from the database for these groups. From 1999 to 2023, excluding the years with suppressed data, there were 328 HDP-related deaths among Hispanic or Latino individuals, 51 among Asian or Pacific Islander Individuals, and 13 among American Indian or Alaska Native individuals (Supplementary Table S6).

### 3.2 Age-group stratified results

Across the study period, 1,058 deaths were recorded for individuals aged 25–34, and 793 deaths among those aged 35–44. The AAMR for individuals aged 25–34 declined throughout the period from 0.12* (95% CI 0.09 to 0.16) in 1999 to 0.11* (95% CI 0.08 to 0.11) in 2023 with an APC and AAPC of −1.24* (95% CI −1.97 to −0.44).

**Figure 3:**
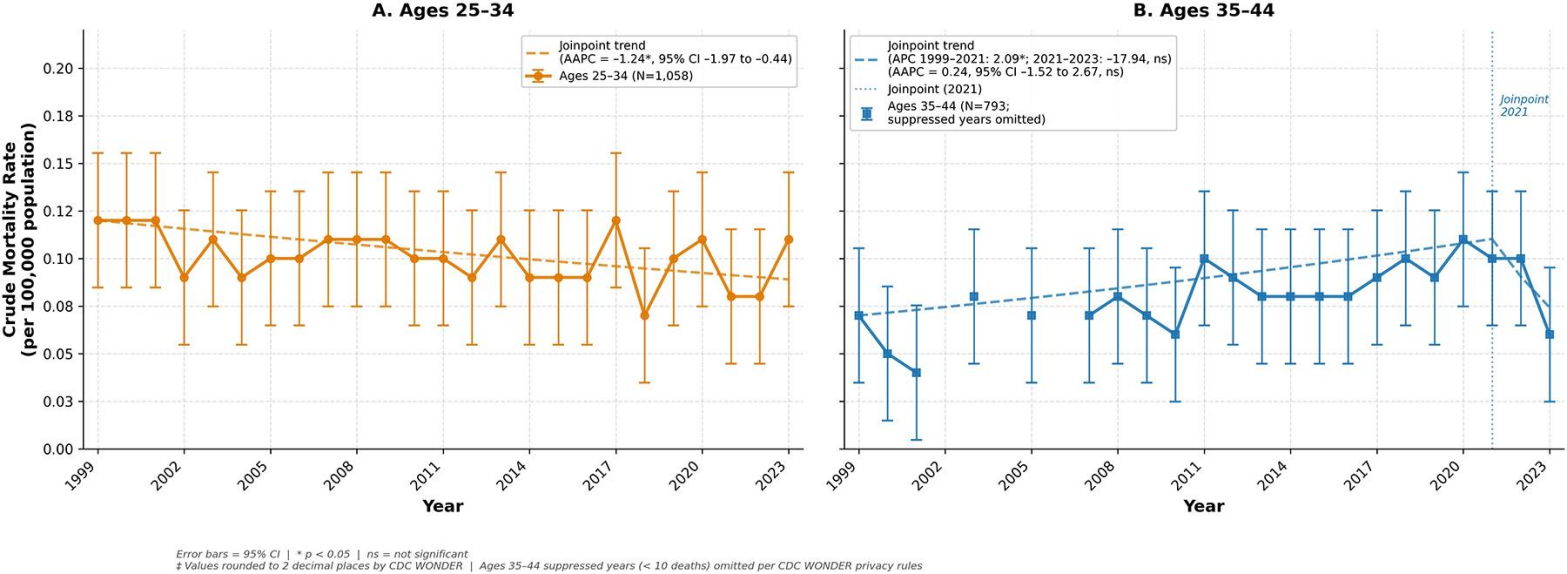
Crude mortality rate from hypertensive disorders of pregnancy by maternal Age group, United States, 1999–2023. Rates are presented per 100,000 population as crude mortality rates. Error bars represent 95% confidence intervals. Dashed lines represent Joinpoint trend lines: Ages 25–34 (AAPC = −1.24*, 95% CI −1.97 to −0.44); Ages 35–44 (AAPC = 0.24, 95% CI −1.52 to 2.67, ns; APC 1999–2021: 2.09*, APC 2021–2023: −17.94, ns). Years with suppressed data (<10 deaths) omitted per CDC WONDER privacy rules. * *p* < 0.05. ns = not significant. Values are rounded to two decimal places as returned by CDC WONDER. AAMR, age-adjusted mortality rate; AAPC, average annual percent change; APC, annual percent change; CI, confidence interval.

In contrast, the AAMR for those aged 35–44 increased throughout the period from 0.07* (95% CI 0.05 to 0.10) in 1999 to 0.10* (95% CI 0.08 to 0.14) in 2021 (APC 2.09*, 95% CI 0.82 to 15.49). This was followed by a nonsignificant decline from 2021 to 2023 (APC −17.94, 95% CI −33.74 to 1.82) (Supplementary Figure S3). Across the full study period, the AAPC for individuals aged 35–44 was 0.24 (95% CI −1.52 to 2.67). These diverging trends resulted in a convergence of crude mortality rate between the two age groups from approximately 2018 onward (Figure 3). Years with unreliable data were removed from the analysis (Supplementary Table S3).

### 3.3 Census region results

**Figure 4:**
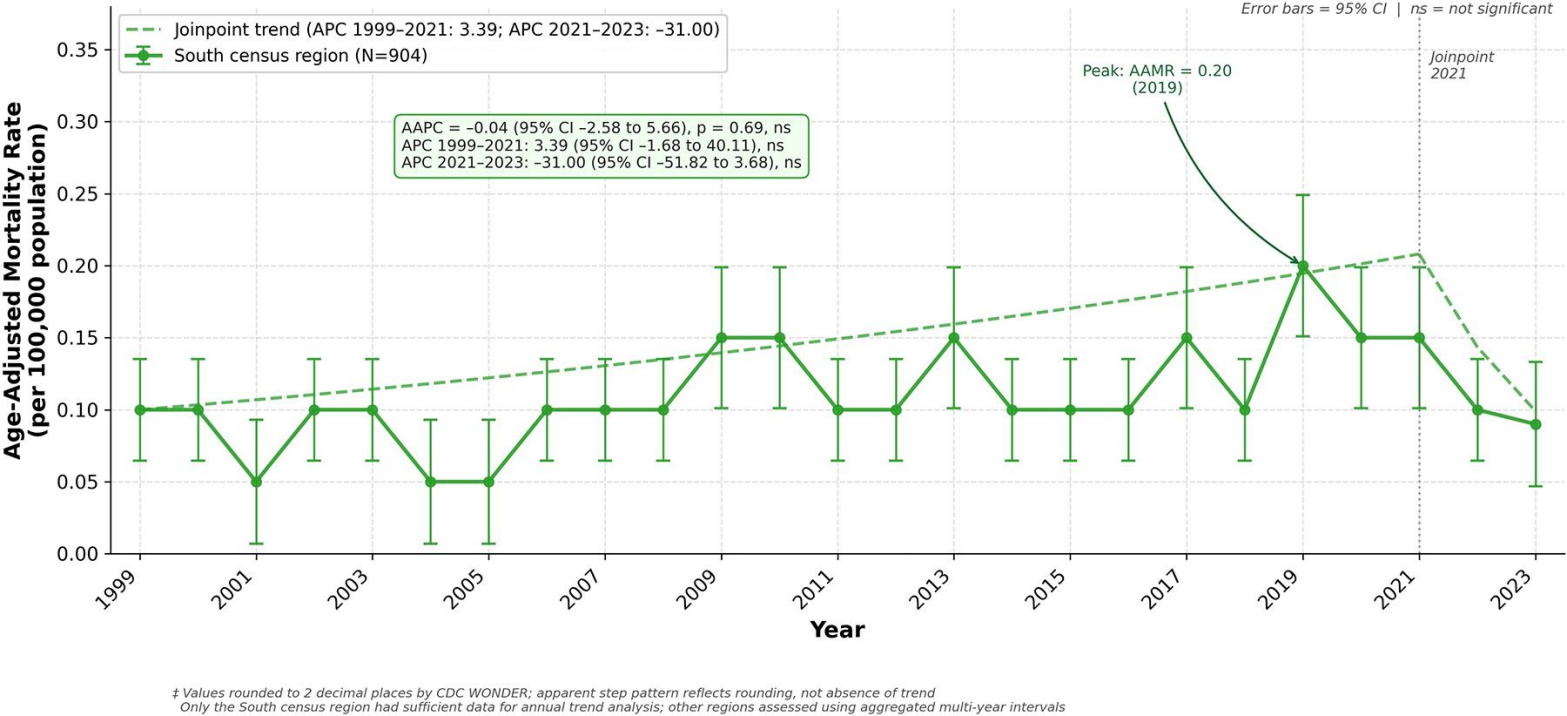
Age-Adjusted mortality rate from hypertensive disorders of pregnancy, south census region, United States, 1999–2023. Rates are presented per 100,000 population and age-adjusted to the 2,000 US standard population. Error bars represent 95% confidence intervals. The dashed line represents the Joinpoint trend line (APC 1999–2021: 3.39, 95% CI −1.68 to 40.11, ns; APC 2021–2023: −31.00, 95% CI −51.82 to 3.68, ns; AAPC = −0.04, 95% CI −2.58 to 5.66, ns). Only the South census region had sufficient data for annual trend analysis. * *p* < 0.05. ns = not significant. Values are rounded to two decimal places as returned by CDC WONDER. AAMR, age-adjusted mortality rate; AAPC, average annual percent change; APC, annual percent change; CI, confidence interval.

There were 904 deaths due to HDP in the South census region between 1999 and 2023. The AAMR increased from 0.10 in 1999 and peaked at 0.20 in 2019 before declining to 0.09 in 2023(Figure 4, Supplementary Table S4). The AAPC was −0.04 (95% CI −2.58 to 5.66) with an APC of 3.39 (95% CI −1.68 to 40.11) from 1999 to 2021 and −31.00 (95% CI −51.82 to 3.68) from 2021 to 2023. Neither segment nor the overall trend reached statistical significance (Supplementary Figure S4).

When deaths were aggregated across the full study period, HDP-related maternal mortality burden was observed across all census regions, with 904 deaths in the South, 323 in the West, 303 in the Midwest, and 283 in the Northeast from 1999 to 2023 (Supplementary Table S7).

### 3.4 Urban-Rural classification results

**Figure 5:**
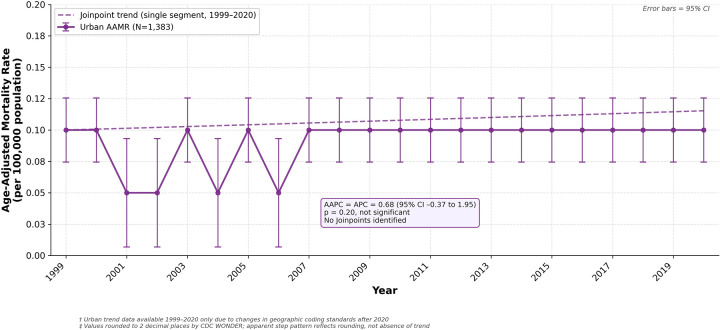
Age-Adjusted mortality rate from hypertensive disorders of pregnancy, urban population zones, United States, 1999–2020. Rates are presented per 100,000 population and age-adjusted to the 2,000 US standard population. Error bars represent 95% confidence intervals. The dashed line represents the Joinpoint trend line (AAPC = APC = 0.68, 95% CI −0.37 to 1.95, ns). Data are limited to 1999–2020 due to changes in geographic coding standards after 2020. * *p* < 0.05. ns = not significant. Values are rounded to two decimal places as returned by CDC WONDER. AAMR, age-adjusted mortality rate; AAPC, average annual percent change; APC, annual percent change; CI, confidence interval.

There were 1,383 deaths due to HDP in urban population zones between 1999 and 2020. While the AAMR was 0.10 in both 1999 and 2020, rates fluctuated between 0.05 and 0.10 from 1999 to 2006 before stabilizing at 0.10 for the remainder of the study period Overall, the AAMR did not change significantly across the study period, with an AAPC and APC of 0.68 (95% CI −0.37 to 1.95) (Figure 5, Supplementary Table S5).

## 4 Discussion

This study highlights that HDP-related maternal mortality has increased over the past two decades, with a consistent upward trend extending through 2023, providing updated insight into recent national trends not fully captured in prior analyses. The use of CDC WONDER enables a comprehensive, longitudinal assessment of maternal mortality patterns across the US, which may not be captured in smaller or institution-specific datasets. These findings add to the existing literature by demonstrating that these trends have remained consistent over time, rather than solely updating prior estimates. Importantly, the absence of significant inflection points suggests that this increase has occurred as a continuous and persistent trend rather than being driven by discrete temporal shifts, suggesting that advances in prenatal monitoring and therapeutic options have not resulted in measurable reductions in HDP-related mortality at the population level (5, 6). In this context, the identification of sustained increases in HDP-related mortality when outcomes were examined by race and age underscores the persistent inequities that continue to shape maternal health outcomes in the US.

NH Black individuals consistently experienced substantially higher and increasing HDP mortality rates compared to NH White individuals. In contrast, mortality among NH White individuals showed no statistically significant overall change, though rates rose between 1999 and 2020 before declining sharply. Previous studies demonstrate that NH Black women are more likely to have their symptoms dismissed, receive inadequate or delayed management of hypertension, and deliver in hospitals with fewer resources and worse maternal outcomes (19, 21). Thus, structural racism, unequal access to quality obstetric care, and delayed recognition and treatment of hypertensive disease in pregnancy may contribute to the disproportionate mortality burden faced by NH Black individuals in this study (21, 22). Although yearly analysis could not be completed for Hispanic or Latino, NH American Indian or Alaska Native, and NH Asian or Pacific Islander individuals due to insufficient data, aggregate mortality counts across the study period demonstrate a persistent mortality impact of HDP-related maternal deaths in these populations. Thus, the absence of yearly specific mortality estimates reflects the dataset and does not demonstrate an absence of risk. Underrepresentation of these groups in national analyses may contribute to delayed recognition of disparities and inadequate prevention efforts (22).

Age-stratified analysis demonstrated shifting patterns in HDP-related mortality, with mortality among individuals aged 25–34 declining and mortality among individuals aged 35–44 increasing, exceeding that of the former age group by the end of the study period. This trend observed in our study may reflect a documented shift in the demographic profile of pregnant women, where delaying pregnancy results in a greater proportion of pregnancies in women older than age 35 (23). Advanced maternal age (AMA) itself contributes to increased HDP-related morbidity and mortality through age-related endothelial dysfunction, increased systemic inflammation, and a higher prevalence of hypertension, diabetes, and obesity (24–27). Thus, the observed increase in HDP-related mortality among individuals aged 35–44 likely reflects both the increasing proportion of pregnancies occurring at an older maternal age as well as the associated age-related physiological risks. Together, these risks underscore the importance of optimizing cardiovascular health prior to contraception and enhancing monitoring throughout pregnancy and the postpartum period, especially for women who are pregnant later in life.

Regional analysis of patients within the South census region showed that HDP-related mortality has gradually increased over time. Chronic hypertension during pregnancy more than doubled in the U.S. from 1.8% to 3.7% between 2008 and 2021, with the highest rates in the South, where HDP affects about 8.9% of pregnancies (25, 26). This regional disparity observed in our study may reflect higher rates of obesity, diabetes, and chronic hypertension, along with limited prenatal and obstetric care access in the South as compared to other regions (2). Declining early prenatal care and widespread maternity care deserts in the South further delay HDP detection and contribute to rising maternal mortality (28, 29). While statistically significant temporal trends were only observed in the South census region, aggregated analyses demonstrated cumulative HDP-related mortality across all U.S. census regions. The presence of a mortality burden in the Northeast, Midwest, and West highlights that HDP-related maternal death is a national issue, even in regions where annual case counts are insufficient to support trend analyses (29). Thus, prevention strategies and resource allocation should not be limited to regions with statistically detectable trends.

Analysis of HDP-related mortality in urban population zones showed no significant change during the study period. Although patients in urban areas continue to experience the burden of hypertensive disorders of pregnancy, increased access to healthcare may have helped prevent further increases in maternal mortality, as urban zones are more likely to have higher concentrations of hospitals, specialty clinics, medical practitioners, and specialists (30). Disparities in access to obstetric care experienced by patients in rural zones increase the need to assess the burden of HDP (31).

These findings are consistent with prior studies using hospitalization and delivery-based datasets. An analysis of the National Inpatient Sample (NIS) demonstrated increasing rates of HDP-related maternal mortality and persistent disparities with reported mortality rates of approximately 14.2 deaths per 100,000 live births, with significantly higher rates among NH Black compared to NH White women (2). In the present study, HDP-related mortality remained at 0.10 per 100,000 population between 1999 and 2023, with a statistically significant upward trend detected by Joinpoint analysis. While absolute rates appear smaller than those reported in studies using live birth denominators, this difference is expected due to the use of population-based rates in this analysis.

Together, these results suggest a potential role for incorporating predictive markers, such as microalbuminuria, elevated systolic blood pressure, and increased serum uric acid levels, into risk stratification efforts, as they are associated with progression to more severe hypertensive disease, such as preeclampsia (32). Integration of these markers alongside demographic and clinical risk factors may help improve early identification of high-risk patients and address persistent disparities present in HDP-related outcomes.

### 4.1 Limitations

This study is limited by the reporting thresholds of the CDC WONDER database, which deems mortality rates as unreliable when the death count is less than twenty individuals that meet the specific search criteria, due to increased standard error (9). Additionally, mortality count is suppressed for privacy when fewer than ten deaths occur within a subgroup during a given period (9). As a result, analysis for certain racial and ethnic groups (NH American Indian or Alaska Native, NH Asian or Pacific Islander, and Hispanic or Latino populations), regions (Northeast, Midwest, West), and rural populations could not be performed due to an insufficient number of HDP-related maternal deaths by year within these populations. Thus, year-to-year comparisons across all demographic groups could not be made, and population trends may underestimate disparities in underrepresented groups. To address this limitation, aggregated mortality counts across the study period were examined to contextualize HDP-related deaths in populations with suppressed annual estimates and characterize the cumulative burden. While aggregation reduces data suppression and improves completeness, it limits temporal resolution and prevents assessment of yearly trends within these subgroups. Additionally, as this is a descriptive analysis, these findings represent associations and do not allow for direct evaluation of underlying mechanisms.

## 5 Conclusion

Overall, HDP poses a threat to maternal health in the US. Despite advancements in prenatal screening and increased maternal health education, HDP-related mortality is still a prevalent issue in the US that disproportionately affects disadvantaged patient populations, reflecting systemic inequities and disparities in access to healthcare. Increasing efforts to expand data collection relating to maternal health, particularly for underrepresented racial groups and rural populations, are essential to understand the impact of access, resources, policy, and prevention efforts on maternal health. This will ultimately help to reduce mortality from HDP nationwide.

## Data Availability

The original contributions presented in the study are included in the article/Supplementary Material, further inquiries can be directed to the corresponding author/s.
