# Supplementary material for: Hypertensive disorders of pregnancy (HDP) mortality in the United States: a CDC WONDER population analysis, 1999–2023
Source: Front Reprod Health. 2026 May 20;8:1816821. doi: 10.3389/frph.2026.1816821 (PMC13230132; doi:10.3389/frph.2026.1816821)
Supplement: Supplementary file 1 [file Datasheet1.docx]

**Supplemental Table 1:**

| Year | Age-Adjusted Mortality Rate |
| --- | --- |
| 1999 | 0.10 |
| 2000 | 0.05 |
| 2001 | 0.05 |
| 2002 | 0.05 |
| 2003 | 0.10 |
| 2004 | 0.05 |
| 2005 | 0.10 |
| 2006 | 0.05 |
| 2007 | 0.10 |
| 2008 | 0.10 |
| 2009 | 0.10 |
| 2010 | 0.10 |
| 2011 | 0.10 |
| 2012 | 0.10 |
| 2013 | 0.10 |
| 2014 | 0.10 |
| 2015 | 0.10 |
| 2016 | 0.10 |
| 2017 | 0.10 |
| 2018 | 0.10 |
| 2019 | 0.10 |
| 2020 | 0.10 |
| 2021 | 0.10 |
| 2022 | 0.10 |
| 2023 | 0.10 |
| Number of Joinpoints (Years of Joinpoint) | 0 |
| APC Segment 1 (95% CI) | 0.89* (0.03 to 1.93) |
| Average APC (AAPC) (95% CI) | 0.89* (0.03 to 1.93) |

**Supplemental Table 1:** **Overall age-adjusted mortality rates from hypertensive disorders of pregnancy, United States, 1999–2023.** Age-adjusted mortality rates are presented per 100,000 population and include joinpoint regression results, annual percent change, and average annual percent change. No joinpoints were identified.

**Supplemental Figure 1:**


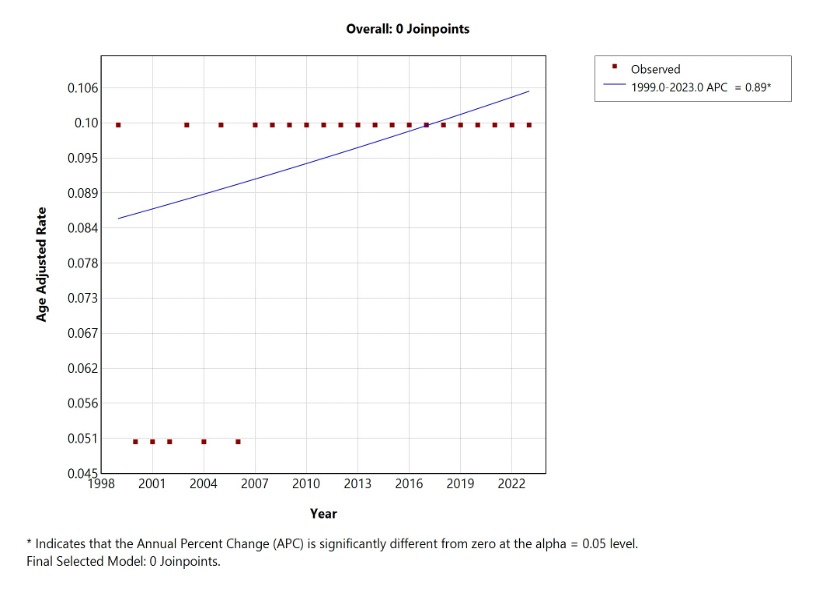


**Supplemental Figure 1: Overall age-adjusted mortality rate from hypertensive disorders of pregnancy, United States, 1999–2023.** Rates are presented per 100,000 population and age-adjusted to the 2000 U.S. standard population. The dashed line represents the joinpoint trend. No joinpoints were identified. AAMR, age-adjusted mortality rate; AAPC, average annual percent change; APC, annual percent change.

**Supplemental Table 2:**

| Year | Age-Adjusted Mortality Rate NH Black or African American | Age-Adjusted Mortality Rate NH White |
| --- | --- | --- |
| 1999 | 0.29 | 0.05 |
| 2000 | 0.19 | 0.05 |
| 2001 | 0.25 | -- |
| 2002 | 0.19 | -- |
| 2003 | 0.35 | 0.05 |
| 2004 | 0.24 | -- |
| 2005 | 0.25 | 0.05 |
| 2006 | 0.19 | -- |
| 2007 | 0.15 | 0.10 |
| 2008 | 0.25 | 0.05 |
| 2009 | 0.29 | 0.05 |
| 2010 | 0.29 | -- |
| 2011 | 0.35 | 0.10 |
| 2012 | 0.40 | -- |
| 2013 | 0.29 | 0.10 |
| 2014 | 0.35 | -- |
| 2015 | 0.31 | 0.05 |
| 2016 | 0.30 | -- |
| 2017 | 0.35 | 0.10 |
| 2018 | 0.25 | 0.10 |
| 2019 | 0.35 | 0.10 |
| 2020 | 0.35 | 0.10 |
| 2021 | 0.35 | -- |
| 2022 | 0.25 | 0.05 |
| 2023 | 0.29 | 0.05 |
| Number of Joinpoints (Years of Joinpoint) | 0 | 1 (2020) |
| APC Segment 1 (95% CI) | 1.52* (0.30 to 2.88) | 3.57* (1.46 to 10.09) |
| APC Segment 2 (95% CI) | -- | -23.13* (-38.36 to -0.56) |
| Average APC (AAPC) (95% CI) | 1.52* (0.30 to 2.88) | -0.22* (-0.22 to -2.81) |

**Supplemental Table 2: Age-adjusted mortality rates from hypertensive disorders of pregnancy stratified by race, United States, 1999–2023.** Age-adjusted mortality rates are presented per 100,000 population for non-Hispanic Black and non-Hispanic White individuals and include Joinpoint regression results, annual percent change, and average annual percent change. One joinpoint was identified among non-Hispanic White individuals in 2020.

**Supplemental Figure 2:**


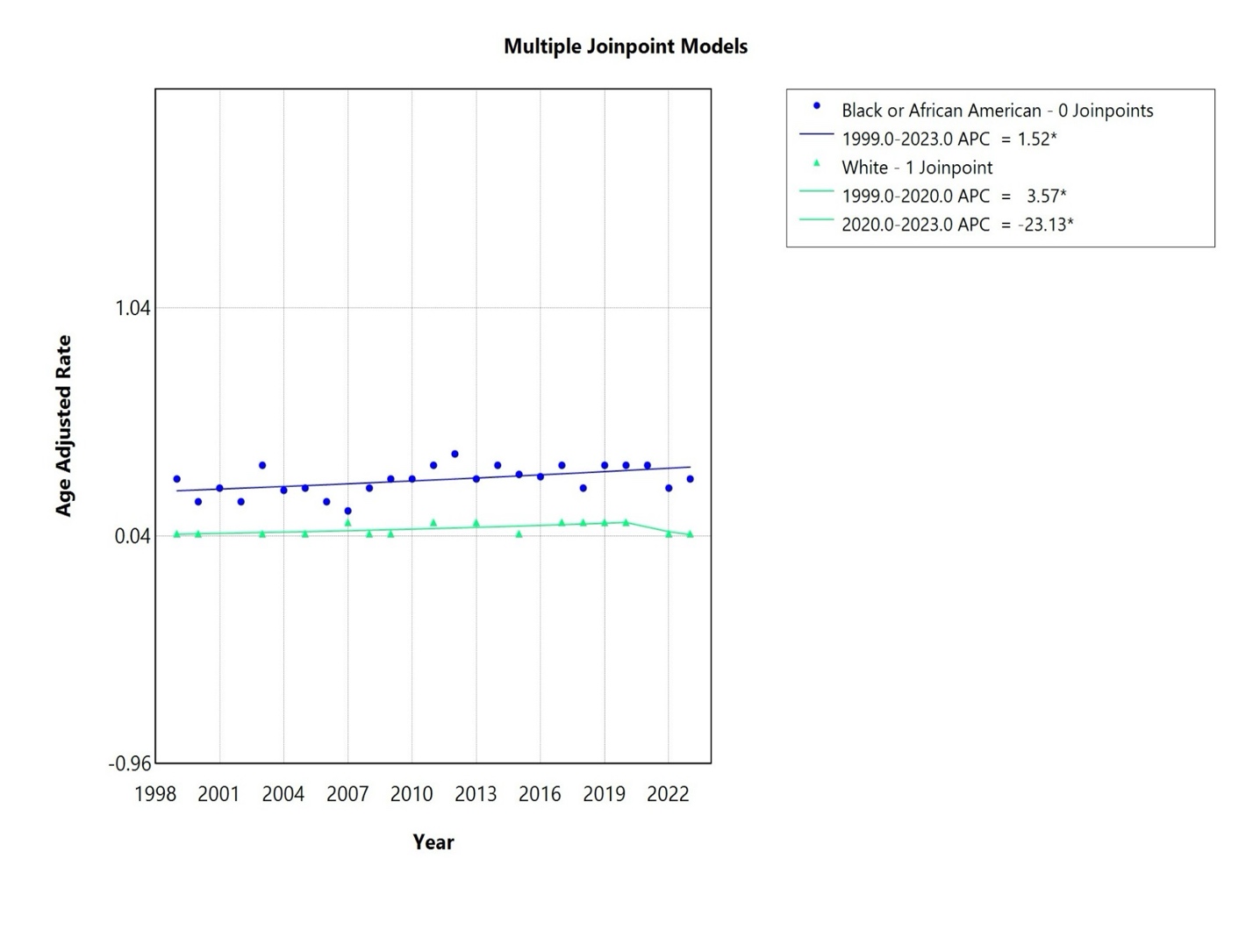


**Supplemental Figure 2:** Joinpoint Model of Hypertensive Disorders of Pregnancy: Age-Adjusted Mortality Rate Stratified by Race, 1999-2023. Rates are presented per 100,000 population and age-adjusted to the 2000 U.S. standard population. Dashed lines represent Joinpoint trends. No joinpoints were identified among non-Hispanic Black individuals, while one joinpoint was identified among non-Hispanic White individuals in 2020. AAMR, age-adjusted mortality rate; AAPC, average annual percent change; APC, annual percent change; NH, non-Hispanic.

**Supplemental Table 3:**

| Year | 25 – 34 Crude Mortality Rate | 35 – 44 Crude Mortality Rate |
| --- | --- | --- |
| 1999 | 0.12 | 0.07 |
| 2000 | 0.12 | 0.05 |
| 2001 | 0.12 | 0.04 |
| 2002 | 0.09 | -- |
| 2003 | 0.11 | 0.08 |
| 2004 | 0.09 | -- |
| 2005 | 0.10 | 0.07 |
| 2006 | 0.10 | -- |
| 2007 | 0.11 | 0.07 |
| 2008 | 0.11 | 0.08 |
| 2009 | 0.11 | 0.07 |
| 2010 | 0.10 | 0.06 |
| 2011 | 0.10 | 0.10 |
| 2012 | 0.09 | 0.09 |
| 2013 | 0.11 | 0.08 |
| 2014 | 0.09 | 0.08 |
| 2015 | 0.09 | 0.08 |
| 2016 | 0.09 | 0.08 |
| 2017 | 0.12 | 0.09 |
| 2018 | 0.07 | 0.10 |
| 2019 | 0.10 | 0.09 |
| 2020 | 0.11 | 0.11 |
| 2021 | 0.08 | 0.10 |
| 2022 | 0.08 | 0.10 |
| 2023 | 0.11 | 0.06 |
| Number of Joinpoints (Years of Joinpoint) | 0 | 1 (2021) |
| APC Segment 1 (95% CI) | -1.24* (-1.97 to -0.44) | 2.09* (0.82 to 15.49) |
| APC Segment 2 (95% CI) | -- | -17.94 (-33.74 to 1.82) |
| Average APC (AAPC) (95% CI) | -1.24* (-1.97 to -0.44) | 0.24 (-1.52 to 2.67) |

**Supplemental Table 3:** **Crude mortality rates from hypertensive disorders of pregnancy stratified by maternal age group, United States, 1999–2023.** Crude mortality rates are presented per 100,000 population for individuals aged 25–34 and 35–44 years and include Joinpoint regression results, annual percent change, and average annual percent change. No joinpoints were identified among individuals aged 25–34 years, while one joinpoint was identified among individuals aged 35–44 years in 2021.

**Supplemental Figure 3:**


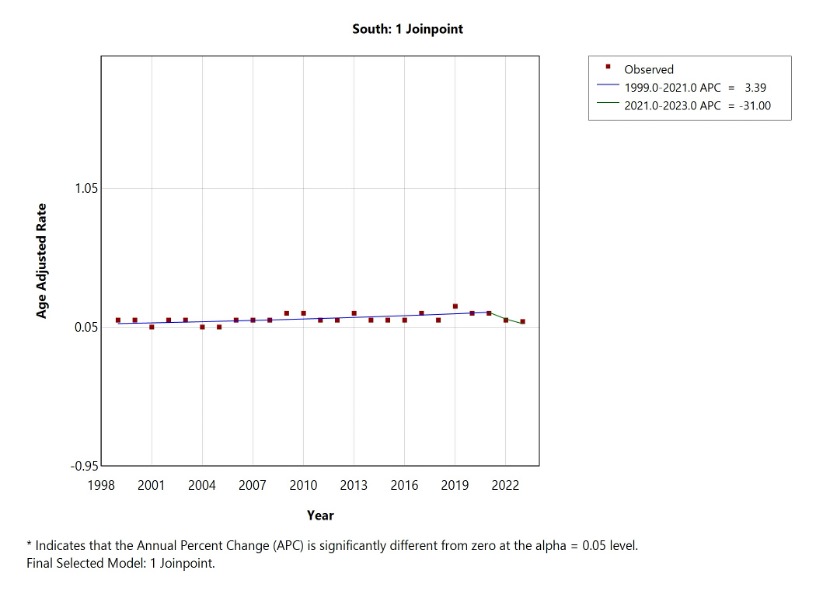


**Supplemental Figure 3: Joinpoint model of crude mortality rates from hypertensive disorders of pregnancy stratified by maternal age group, United States, 1999–2023.** Rates are presented per 100,000 population for individuals aged 25–34 and 35–44 years. Dashed lines represent Joinpoint trends. No joinpoints were identified among individuals aged 25–34 years, while one joinpoint was identified among individuals aged 35–44 years in 2021. AAPC, average annual percent change; APC, annual percent change.

**Supplemental Table 4:**

| Year | Age-Adjusted Mortality Rate |
| --- | --- |
| 1999 | 0.10 |
| 2000 | 0.10 |
| 2001 | 0.05 |
| 2002 | 0.05 |
| 2003 | 0.10 |
| 2004 | 0.05 |
| 2005 | 0.05 |
| 2006 | 0.10 |
| 2007 | 0.10 |
| 2008 | 0.10 |
| 2009 | 0.15 |
| 2010 | 0.15 |
| 2011 | 0.10 |
| 2012 | 0.10 |
| 2013 | 0.15 |
| 2014 | 0.10 |
| 2015 | 0.10 |
| 2016 | 0.10 |
| 2017 | 0.15 |
| 2018 | 0.10 |
| 2019 | 0.20 |
| 2020 | 0.15 |
| 2021 | 0.15 |
| 2022 | 0.10 |
| 2023 | 0.09 |
| Number of Joinpoints (Years of Joinpoint) | 1 (2021) |
| APC Segment 1 (95% CI) | 3.39 (-1.68 to 40.11) |
| APC Segment 2 (95% CI) | -31.00 (-51.83 to 3.68) |
| Average APC (AAPC) (95% CI) | -0.04 (-2.58 to 5.66) |

**Supplemental Table 4: Age-adjusted mortality rates from hypertensive disorders of pregnancy in the South Census Region, United States, 1999–2023.** Age-adjusted mortality rates are presented per 100,000 population and include Joinpoint regression results, annual percent change, and average annual percent change. One joinpoint was identified in 2021.

**Supplemental Figure 4:**


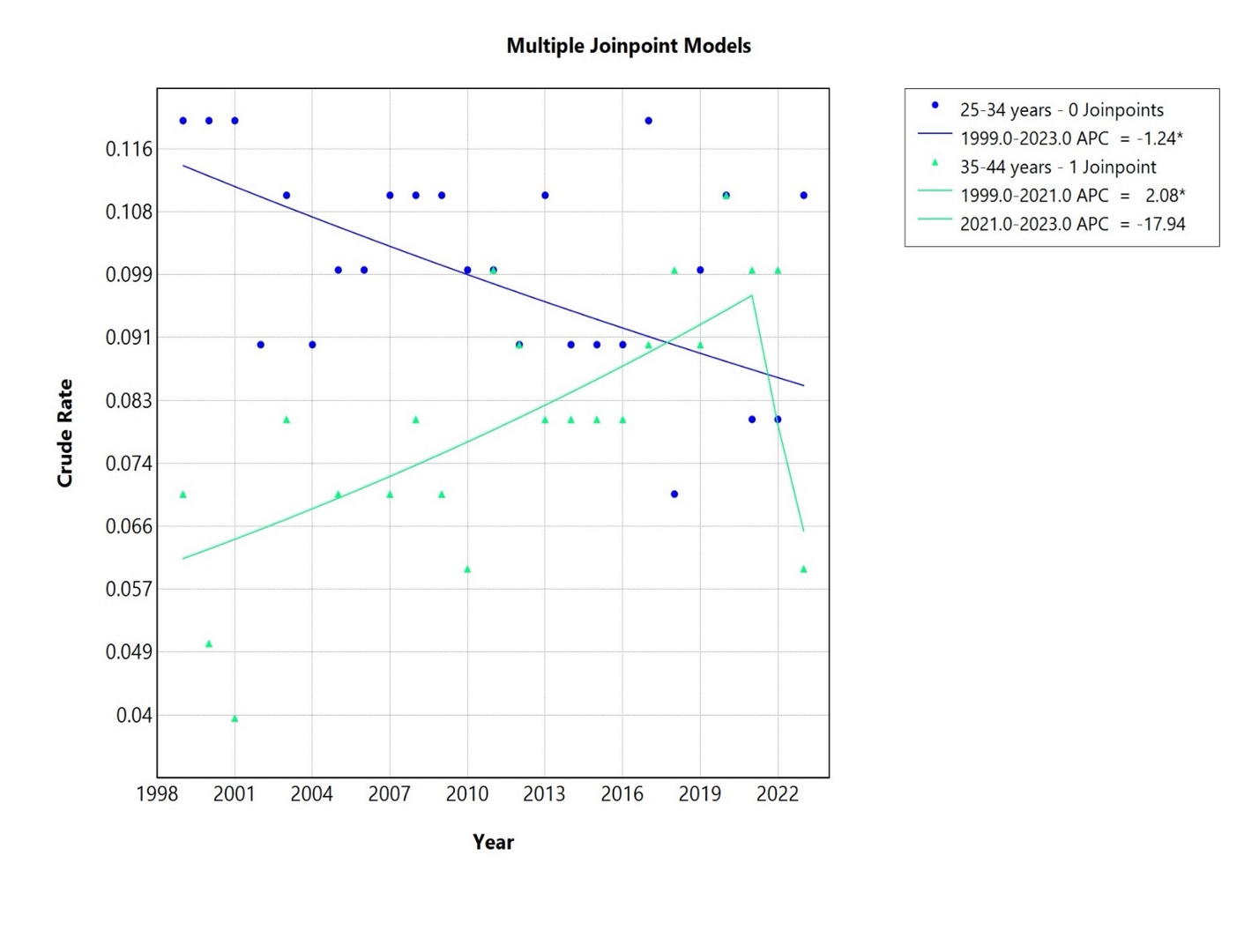


**Supplemental Figure 4: Joinpoint model of age-adjusted mortality rates from hypertensive disorders of pregnancy in the South Census Region, United States, 1999–2023.** Rates are presented per 100,000 population and age-adjusted to the 2000 U.S. standard population. The dashed line represents the Joinpoint trend. One joinpoint was identified in 2021. AAMR, age-adjusted mortality rate; AAPC, average annual percent change; APC, annual percent change.

**Supplemental Table 5:**

| Year | Age-Adjusted Mortality Rate |
| --- | --- |
| 1999 | 0.10 |
| 2000 | 0.10 |
| 2001 | 0.05 |
| 2002 | 0.05 |
| 2003 | 0.10 |
| 2004 | 0.05 |
| 2005 | 0.10 |
| 2006 | 0.05 |
| 2007 | 0.10 |
| 2008 | 0.10 |
| 2009 | 0.10 |
| 2010 | 0.10 |
| 2011 | 0.10 |
| 2012 | 0.10 |
| 2013 | 0.10 |
| 2014 | 0.10 |
| 2015 | 0.10 |
| 2016 | 0.10 |
| 2017 | 0.10 |
| 2018 | 0.10 |
| 2019 | 0.10 |
| 2020 | 0.10 |
| Number of Joinpoints (Years of Joinpoint) | 0 |
| APC Segment 1 (95% CI) | 0.68 (-0.37 to 1.95) |
| Average APC (AAPC) (95% CI) | 0.68 (-0.37 to 1.95) |

**Supplemental Table 5: Age-adjusted mortality rates from hypertensive disorders of pregnancy in urban population zones, United States, 1999–2020.** Age-adjusted mortality rates are presented per 100,000 population and include Joinpoint regression results, annual percent change, and average annual percent change.

**Supplemental Figure 5:**


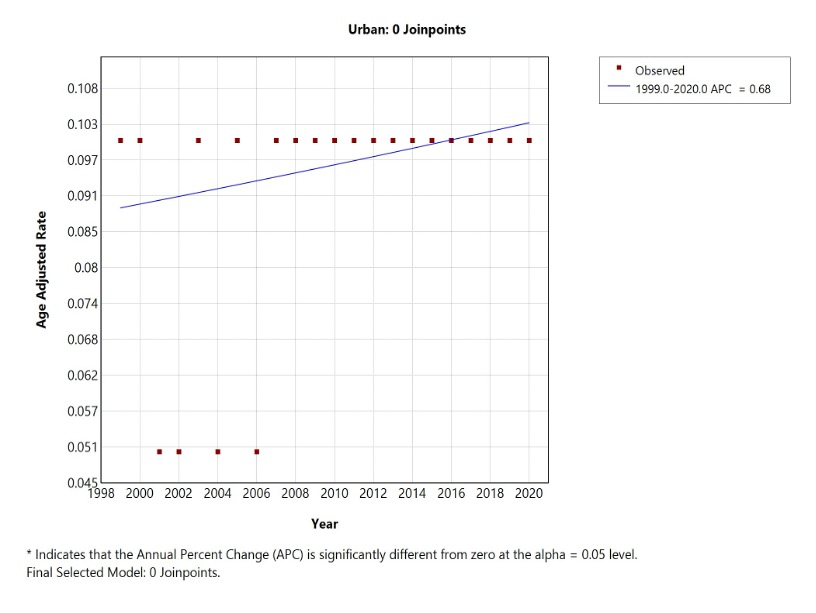


**Supplemental Figure 5: Joinpoint model of age-adjusted mortality rates from hypertensive disorders of pregnancy in urban population zones, United States, 1999–2020.** Rates are presented per 100,000 population and age-adjusted to the 2000 U.S. standard population. The dashed line represents the Joinpoint trend. No joinpoints were identified. AAMR, age-adjusted mortality rate; AAPC, average annual percent change; APC, annual percent change.

**Supplemental Table 6:**

| Race | Deaths during 1999-2020 | Deaths during 2021-2023 | Total deaths |
| --- | --- | --- | --- |
| Non-Hispanic Black | 695 | 105 | 800 |
| Non-Hispanic White | 561 | 77 | 738 |
| Hispanic or Latino | 287 | 41 | 328 |
| Asian | 51 | Suppressed | 51 |
| Native Hawaiian or Other Pacific Islander |  | 0 |  |
| American Indian or Alaska Native | 13 | Suppressed | 13 |

**Supplemental Table 6: Aggregated hypertensive disorder of pregnancy-related maternal deaths by race and ethnicity, United States, 1999–2023.** Deaths are presented for 1999–2020 and 2021–2023, with total deaths across the full study period. Suppressed values indicate categories with counts below reporting thresholds.

**Supplemental Table 7:**

| Census Region | Deaths during 1999-2020 | Deaths during 2021-2023 | Total deaths |
| --- | --- | --- | --- |
| Northeast | 283 | 23 | 306 |
| Midwest | 266 | 45 | 311 |
| South | 771 | 133 | 904 |
| West | 293 | 37 | 330 |

**Supplemental Table 7: Aggregated hypertensive disorder of pregnancy-related maternal deaths by Census region, United States, 1999–2023.** Deaths are presented by U.S. Census region for 1999–2020 and 2021–2023, with total deaths across the full study period.
